# From thunder to silence: Cochlear implantation for bilateral profound sensorineural hearing loss after lightning strike

**DOI:** 10.1007/s00405-026-10495-3

**Published:** 2026-08-07

**Authors:** Asimakis D. Asimakopoulos, Louis Noël, Kishore Sandu, Pascal Senn

**Affiliations:** 1 Department of Otorhinolaryngology - Head and Neck Surgery, Lausanne University Hospital, University of Lausanne, Rue du Bugnon 46, Lausanne, 1011 Switzerland; 2 Department of Otorhinolaryngology - Head and Neck Surgery, Saint- Etienne University Hospital, Saint-Etienne, France; 3 Jacques Lisfranc School of Medicine, University Jean Monnet, Saint- Etienne, France; 4 Department of Otorhinolaryngology - Head and Neck Surgery, Geneva University Hospitals, Geneva, Switzerland; 5 Department of Otorhinolaryngology - Head and Neck Surgery, Jacques Lisfranc School of Medicine, Saint-Etienne University Hospital - Hôpital Nord, University Jean Monnet, Av. Albert Raimond 42270, Saint-Etienne, Saint-Priest-En-Jarez, France

**Keywords:** Lightning strike, Sensorineural hearing loss, Perilymphatic fistula, Cochlear implantation, Vestibular dysfunction

## Abstract

**Introduction:**

Lightning strikes can cause diverse neuro-otologic injuries. Tympanic membrane rupture is common, whereas bilateral profound sensorineural hearing loss with vestibular dysfunction and perilymphatic fistula is rare. Cochlear implantation for lightning-induced deafness has been reported only once.

**Case Summary:**

A farm worker struck by lightning sustained second-degree burns and intracranial hemorrhages requiring emergency neurosurgical management and cervical wound closure. During intensive care, clear left-sided otorrhea prompted ENT evaluation, revealing bilateral tympanic membrane perforations. Beta-trace testing confirmed cerebrospinal fluid leakage. Temporal bone CT demonstrated left ossicular chain dislocation and stapes footplate fracture with perilymphatic fistula, managed by exploratory tympanotomy with fistula closure and tympanic reconstruction. The patient remained with bilateral profound sensorineural hearing loss and left vestibular hypofunction. Six months after injury, right-sided cochlear implantation with a MED-EL Synchrony Flex 28 electrode array was performed. Four months post-implantation, aided thresholds averaged 30 dB HL with 30%-word recognition score at 65 dB SPL. Finally, 14 months after the trauma, left-sided cochlear implantation using the same model was undertaken. Although auditory recovery was slower than on the right side, the patient achieved open-set disyllabic word recognition score of 30% at 65 dB SPL by 3 months post-implantation.

**Discussion:**

This case highlights the destructive potential of lightning on the auditory and vestibular systems and demonstrates that cochlear implantation can provide effective auditory rehabilitation when cochlear patency is preserved.

## Introduction

Lightning strikes cause hundreds of fatalities and several thousands of nonfatal injuries worldwide each year. They are a rare but well-recognized cause of neuro-otologic injury, with the auditory system particularly vulnerable to both direct electrical and blast effects [1, 2]. Tympanic membrane rupture and transient conductive hearing loss occur in more than half of lightning victims and represent the most frequently reported otologic sequelae, whereas severe sensorineural hearing loss (SNHL) and vestibular dysfunction are far less common [3, 4].

Histopathologic studies of temporal bones from lightning victims have demonstrated rupture of Reissner’s membrane, degeneration of the stria vascularis and organ of Corti, edema of the facial nerve, and occasional microfractures of the otic capsule [5, 6]. Proposed mechanisms of injury include direct electrical conduction through cerebrospinal fluid pathways, explosive barotrauma from the lightning shock wave, and thermal injury to cochlear structures [3, 7]. Only a handful of cases of bilateral severe SNHL following a lightning strike have been documented in the literature [3, 8].

We present a case of lightning injury resulting in bilateral profound SNHL, left-sided vestibular deficit, and a traumatic perilymphatic fistula requiring surgical repair, successfully rehabilitated with bilateral cochlear implantation (CI). This case adds to the limited literature on CI after lightning injury, for which, to our knowledge, only a single prior case has been documented [9].

## Case report

A farm worker was admitted to a tertiary care hospital after being struck by lightning while working in a field. The electrical current entered through the occipital region and exited through the left heel. He was resuscitated at the scene and transported by helicopter while intubated because of agitation and a Glasgow Coma Scale score of 12. Physical examination revealed second-degree burns involving approximately 10% of the total body surface area, including a cervical wound at the site of a necklace. Computed tomography of the head demonstrated a right lenticular intraparenchymal hemorrhage and a left frontoparietal subarachnoid hemorrhage. The patient underwent right frontal intracranial pressure monitor placement and surgical debridement with closure of the cervical wound in an emergency setting.

During intensive care monitoring, otorrhea from the left ear was observed while the patient remained intubated, prompting otolaryngologic evaluation. The otoscopy revealed a subtotal right tympanic membrane perforation and a complete left perforation with Beta-trace protein positive for cerebrospinal fluid leakage. The computed tomography of the temporal bone demonstrated a complete dislocation of the left ossicular chain with a fracture of the stapes footplate and a perilymphatic fluid leak, without evidence of another temporal bone fracture. An exploratory tympanotomy confirmed the dislocation of the incudomalleolar and incudostapedial joints and identified a perilymphatic fistula caused by the stapes footplate fracture. The fistula was sealed with tragal perichondrium, bone paté, and cartilage, and the tympanic membrane was reconstructed with an autologous cartilage graft.

The patient was extubated five days after the incident and reported bilateral hearing loss and vertigo. Examination showed a clean right-sided subtotal tympanic membrane perforation, right-beating nystagmus, and an abnormal head impulse test on the left. Pure-tone audiometry demonstrated bilateral profound SNHL, with no measurable responses up to 120 dB HL bilaterally. He subsequently underwent neurological rehabilitation for the intracranial hemorrhages.

At three-month follow-up, bilateral profound SNHL persisted, with left-sided vestibular hypofunction and preserved right vestibular function. Video head impulse testing showed horizontal semicircular canal gains of 0.53 on the left and 1.22 on the right. The left neotympanum graft remained intact, and the right-sided subtotal perforation was clean. Repeat computed tomography and magnetic resonance imaging revealed no evidence of cochlear ossification.

**Fig. 1:**
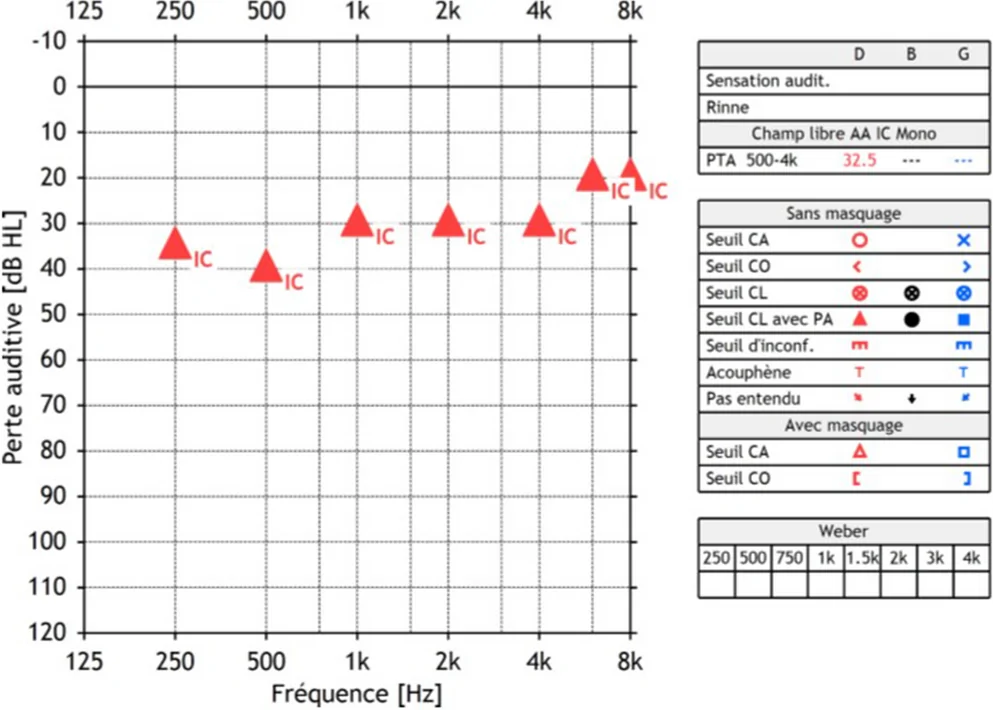
Pure-Tone Audiometry Results of the Right ear. Pure-tone audiogram performed four months after right cochlear implantation demonstrating aided air-conduction thresholds averaging approximately 30 dB HL across 0.5–4 kHz in the implanted ear, consistent with effective auditory rehabilitation

**Fig. 2:**
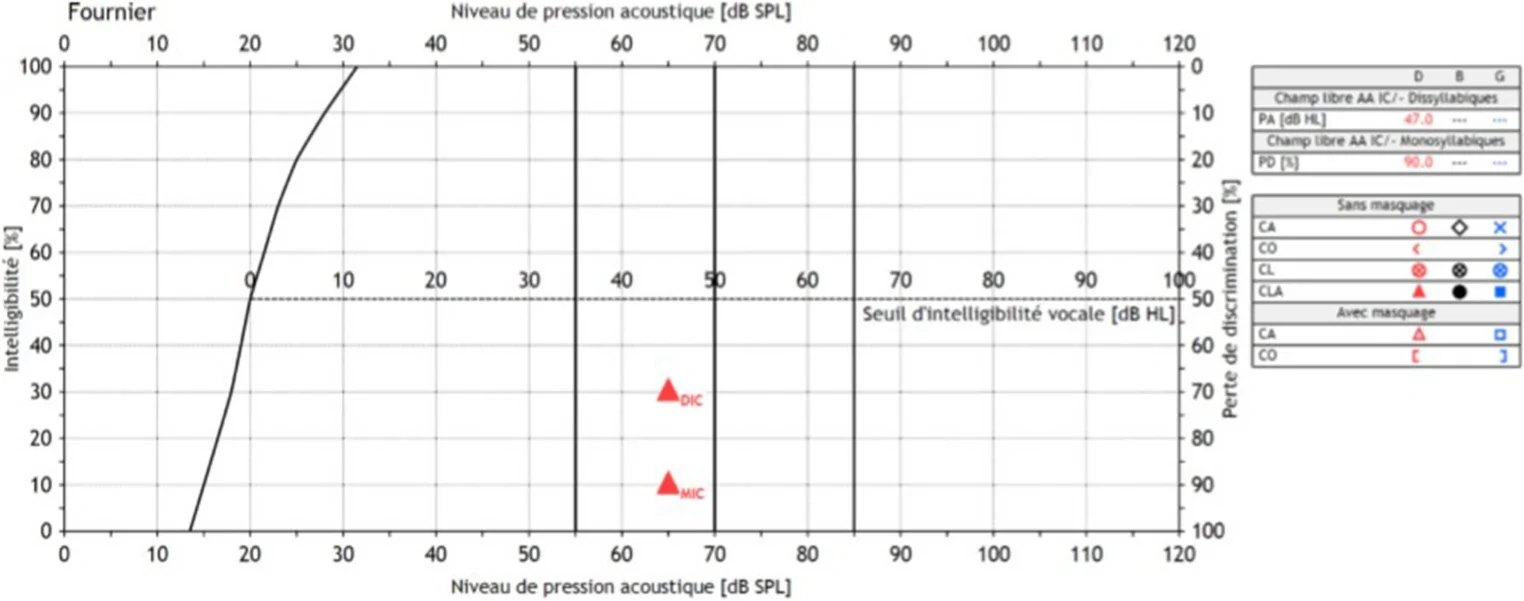
Postoperative Speech Audiometry Results of the Right ear. Speech audiometry performed four months after right cochlear implantation using French Fournier open-set monosyllabic and disyllabic word lists

**Fig. 3:**
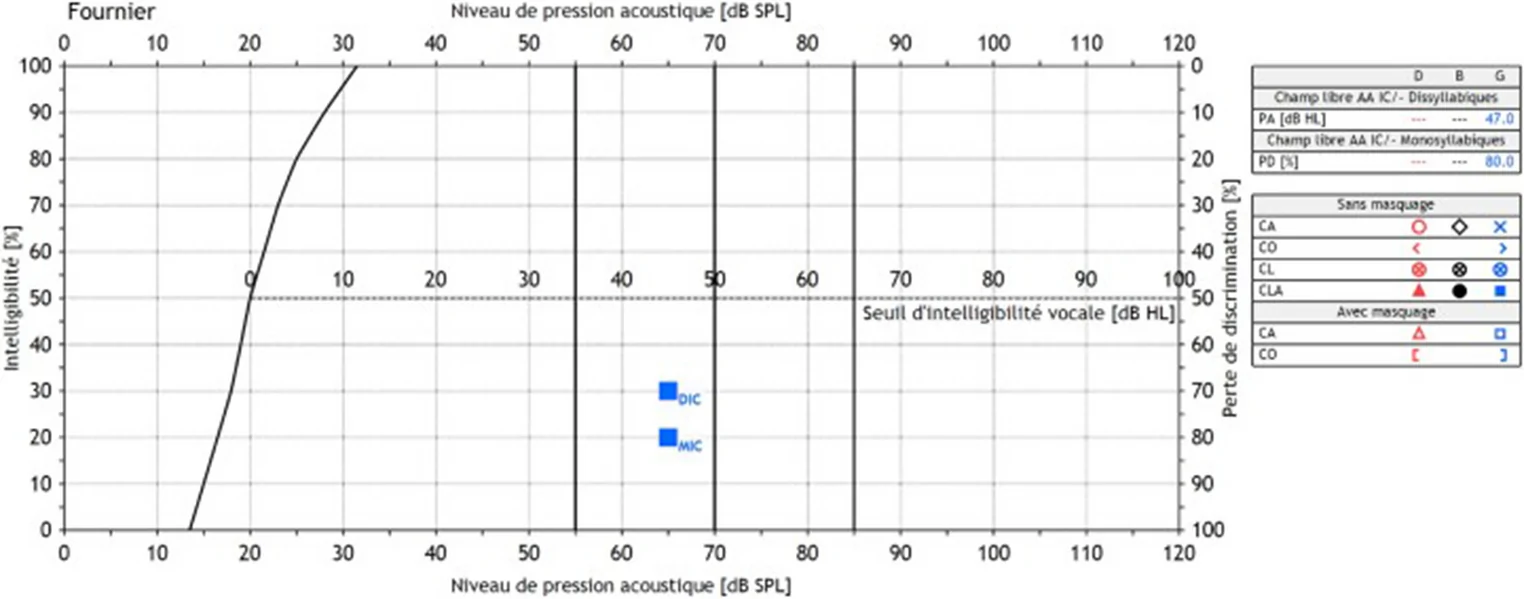
Postoperative **S**peech Audiometry of the Left ear. Speech audiometry performed nine months after left cochlear implantation using French Fournier open-set monosyllabic and disyllabic word lists

Six months after the lightning injury, the patient underwent right-sided CI with a MED-EL Synchrony Flex 28 electrode array (MED-EL, Innsbruck, Austria), accompanied by right-sided tympanoplasty using a temporalis fascia graft. Given the absence of measurable auditory responses up to 120 dB HL bilaterally, a trial of conventional amplification was not pursued, as patients with profound SNHL at this level are known to derive minimal to no functional benefit from hearing aids, with CI representing the standard rehabilitative option in such cases. Four months postoperatively, pure-tone audiometry (Fig. 1) demonstrated aided thresholds averaging 30 dB HL across 0.5–4 kHz, and speech audiometry (Fig. 2) showed a word-recognition score of 30% at 65 dB SPL. Encouraged by these favorable outcomes, the patient requested CI of the more affected contralateral ear, which had previously undergone perilymphatic fistula repair. The implantation was carried out 14 months after the lightning injury using the same MED-EL Synchrony Flex 28 system. Despite the history of stapes footplate fracture and perilymphatic fistula repair, no evidence of cochlear ossification, fibrosis, or abnormal anatomy was encountered intraoperatively. Full electrode insertion was achieved through a standard round-window approach without surgical difficulty. Auditory recovery on the left side progressed more slowly in the early postoperative period compared with the right side but achieved open-set disyllabic word recognition score of 30% at 65 dB SPL by 3 months post-implantation (Fig. 3).

Postoperative speech audiometry should be interpreted with caution in this patient. The recorded scores were obtained using French Fournier open-set monosyllabic and disyllabic word lists, whereas French was not the patient’s native language. Speech audiometry is ideally performed in the patient’s native language or with linguistically familiar material, as lexical familiarity and language proficiency can significantly influence open-set word-recognition performance. This issue may be even more relevant in cochlear implant users, in whom speech-recognition outcomes show substantial variability and are sensitive to linguistic and cognitive factors. Therefore, the formal postoperative speech scores may have underestimated the patient’s real-world auditory benefit. Consistent with this, despite only modest scores on French word testing, the patient was able to communicate effectively with family members in everyday situations without relying exclusively on lip-reading.

Aided hearing thresholds for the left cochlear implant were not obtained. Following the second cochlear implantation, the patient permanently returned to his home country, resulting in limited availability for long-term follow-up. Consequently, comprehensive audiological and vestibular follow-up assessments could not be performed at our institution.

Nine months after the second cochlear implantation, the patient underwent a complete vestibular assessment. Video head-impulse testing (vHIT) demonstrated markedly reduced vestibulo-ocular reflex (VOR) gains of the left anterior (0.40), lateral (0.10), and posterior (0.11) semicircular canals, with normal right-sided gains (anterior 0.95, lateral 1.02, posterior 0.70), consistent with left-sided three-canal hypofunction. Cervical and ocular vestibular-evoked myogenic potentials (cVEMP and oVEMP) showed present responses on the right and absent responses on the left, indicating loss of ipsilateral saccular and utricular function. Taken together, these findings document chronic, unilateral left vestibular failure with preserved contralateral function, in the setting of stable bilateral cochlear implant performance.

## Discussion

This case presentation highlights the therapeutic efficacy of CI in the context of severe inner ear trauma with SNHL, vestibular injury and middle ear barotrauma in a survivor of a lightning strike. The clinical picture of the presented patient is consistent with the most severe spectrum of lightning-related inner-ear injury. The tympanic membrane perforation remains the most frequently encountered otologic manifestation, generally attributed to the cylindrical blast wave generated in the external auditory canal [5, 10]. However, the absence of temporal bone fracture and the presence of severe cochlear dysfunction in this case could support direct electrical conduction as one of the primary mechanisms, in which current may travel through cerebrospinal fluid pathways toward the internal auditory canal, predominantly affecting cochlear hair cells, while preservation of the cochlear nerve is suggested by the favorable outcomes following CI [5, 6]. Severe acoustic trauma can induce a severe SNHL and might be of one the mechanisms involved in our patient as well.

Perilymphatic fistula caused by stapes footplate fracture is exceedingly rare after lightning exposure; only a few isolated cases of lightning-related perilymphatic fistulas have been documented, including bilateral perilymphatic fistulas and a case associated with facial palsy [8, 11]. Comparable reports of profound bilateral SNHL following electrocution or lightning strike remain limited to small case series and single reports. Mora-Magaña et al. [7] described otologic trauma with mixed hearing loss following lightning exposure, whereas Goodwin et al. [6] and Choi et al. [12] documented sensorineural deficits after high-voltage electrical injury. Offiah et al. [10] reported the first case of ossicular-chain disruption secondary to a lightning strike, underscoring the wide spectrum of inner-ear and middle-ear pathologies associated with lightning trauma.

The choice to perform CI on the right side first warrants consideration. Although the left ear presented with a stapes footplate fracture and associated perilymphatic fistula—conditions that may raise concern for subsequent intracochlear fibrosis or ossification—several factors supported prioritization of the right ear. Notably, the patient reported pre-existing hearing impairment on the left side prior to the lightning injury, suggesting a less favorable baseline auditory substrate. In addition, the perilymphatic fistula on the left was surgically repaired with successful sealing and without postoperative complications or signs of infection, and follow-up imaging did not demonstrate cochlear ossification. Therefore, the right ear was considered to offer a more predictable auditory outcome for initial implantation. Following favorable results, sequential implantation of the contralateral ear was subsequently performed.

Our patient’s management aligns closely with the only published case of CI after lightning injury reported by Myung et al. [9]. In that case, a 45-year-old woman achieved excellent speech perception after right CI nine months post-strike. Similar to their findings, our patient showed no cochlear ossification on imaging and obtained robust postoperative open-set speech perception.

## Conclusion

Lightning strikes can inflict catastrophic auditory injury, including irreversible bilateral SNHL. Survivors presenting with tympanic membrane perforation, otorrhea, or sudden hearing loss warrant prompt neuro-otologic assessment and imaging to exclude temporal bone fracture, and early repair of perilymphatic fistulas when present. In cases of irreversible bilateral profound SNHL, CI should be considered without delay. This case highlights both the destructive potential of lightning on the auditory system and the efficacy of CI as an auditory rehabilitative strategy for lightning-induced severe SNHL.
